# An Implementation Gap of 120 Years: Complex Problems, Such as Family Planning, Pregnancy, and Parenthood in Surgical Training Require Suitable Research Paradigms and Innovative Change

**DOI:** 10.1002/wjs.12553

**Published:** 2025-03-11

**Authors:** Rhea Liang

**Affiliations:** ^1^ Bond University Faculty of Health Sciences and Medicine Gold Coast Australia; ^2^ Gold Coast Hospital and Health Service Southport Australia

Doctors are trained in the techniques of biomedical science, which have led to incredible breakthroughs. However, this research paradigm can also be a weakness, because biomedical research techniques aim to minimize variability and exclude statistical outliers.

This matters a great deal in sociological phenomena such as the lack of diversity in the surgical workforce. When applied through individual implicit biases and further constrained by existing systemic biases, biomedical research techniques risk exclusion, such as the omission of women as “outliers,” from definitions of the qualities of a good surgeon [1]. By smoothing the ripples and richness of “variability,” a biomedical approach also risks overgeneralization, such as stereotyping of women as the “causative factor” in a higher rate of choosing to leave surgical training [2].

Compounding these pitfalls, doctors have been late to embrace complexity and develop skills to translate research findings into effective action in an often messy world. The time lag between evidence and implementation is said to be 17 years, but for women in medicine, it is already far longer. The first woman surgeon in Australia was Lilian Violet Cooper, who became a Fellow of the Royal Australasian College of Surgeons on its founding in 1928. She had been one of the first to publish about gender bias, writing in 1904

Till very recent years the environment of the woman student in any branch of science has been so persistently unfavourable as to be entirely prohibitive of any material advancement. Arguments as rancorous as they were unscientifically based and superstitious were used against the girl‐scholar, whose only aim was enlightenment and a not unnatural desire to achieve something of that social independence and oft as not professional success, which fitting education alone can render possible.

Ref. [3]

In this issue, more than 120 years later, Xu et al. continue the work toward a “fitting education” in three important ways [4]. Firstly, by studying all genders and not just women, Xu et al. take an inclusive stance and incorporate current thinking about complexity and universal design for learning, often described as the “rising tide lifts all boats” approach. For example, concerns about high rates of women choosing to leave surgical training has resulted in beneficial changes to address fatigue and wellness for all trainees not just women. Similarly, measures originally designed to support maternity leave have evolved into parental leave for all genders and even further to flexible training for any reason—something particularly salient post‐COVID with high rates of an unplanned leave across the surgical workforce [5].

Secondly, studying all genders also mitigates against researcher bias by moving away from a narrative where men are the norm and women the “other.” For example, the double standard between “valid” and “invalid” reasons for leave has been previously noted, with absence for reasons, such as miscarriage or pregnancy, treated very differently to equivalent periods of absence for musculoskeletal injuries or research [2]. Qualitative data in Table 2 from Xu et al. contain examples of the lack of support for regaining surgical skills after parental leave and formal complaints lodged against breastfeeding at work. It would seem unthinkable for equivalent discriminations against a trainee returning to clinical practice after an injury or sanctions for finishing “academic” paper revisions in a “clinical” workplace. By problematizing these phenomena, Xu et al. contribute to a more equitable narrative where actions to achieve diversity do not require “fixing women” but become a shared effort for everyone.

Thirdly, Xu et al. take advantage of the unique Australasian setting to conduct research in arguably the most contained surgical training environment globally. As detailed in the paper, Australasia has one college covering the majority of surgical specialties, the Royal Australasian College of Surgeons (RACS). The College combines the functions of multiple organizations in comparable health systems. It governs the delivery of surgical training, confers the Fellowship which is required for specialist registration, and sets the standards expected for continued professional practice. In effect, research in the RACS environment is a geographically defined experiment across the full range of surgical practice contexts—metropolitan to remote, ambulatory and inpatient, and public and private—in a population of approximately 1200 trainees at any given time. The findings from Xu et al. should therefore be widely transferable to many surgical training locations worldwide.

Where to from here? Echoing the call of Vasey et al. [5], Xu et al. frame family planning, pregnancy, and parenthood as a systemic not individual challenge. They call for structural changes to assist return to work for any reason, citing the example of the recently published *Returning to work after a period of leave* policy from RACS [6]. They argue for universally available flexible work options and sufficient support for clinical service delivery around the parental leave period.

These suggestions have parallels in the United States, United Kingdom, and elsewhere; but the key shift is to no longer consider this of primary benefit to women trainees—which might be considered benevolent sexism—but to acknowledge the benefits to everyone. If surgical training were truly a “leaky pipeline,” one might expect everyone to fall out of the leaks at the same rate. The overwhelming evidence that the “pipeline” preferentially leaks women and other underrepresented groups suggests surgical training is more like a sieve with holes of only one size and shape.

Xu et al. contribute to a growing narrative that the remedy for this is not focusing narrowly on women, or any other group, but to find universally inclusive solutions based on a shared mission of surgical excellence. To do this requires a move beyond familiar biomedical research paradigms and to resist defining ingroups and outgroups based on historical norms, which recreate existing systemic bias through experimental design. Surgeons embody *par excellence* the ability to work with complexity, to adopt new paradigms, and to innovate on available information even if it is a departure from known practice. Let us now apply those skills to the challenge of achieving equity in the workforce and for the diverse communities we serve.

## Author Contributions

**Rhea Liang:** conceptualization, writing – original draft.
